# Pathways to increased coverage: an analysis of time trends in contraceptive need and use among adolescents and young women in Kenya, Rwanda, Tanzania, and Uganda

**DOI:** 10.1186/s12978-017-0393-3

**Published:** 2017-10-17

**Authors:** Mardieh L. Dennis, Emma Radovich, Kerry L. M. Wong, Onikepe Owolabi, Francesca L. Cavallaro, Michael T. Mbizvo, Agnes Binagwaho, Peter Waiswa, Caroline A. Lynch, Lenka Benova

**Affiliations:** 1 Faculty of Epidemiology & Population Health, London School of Hygiene & Tropical Medicine, Keppel Street, London, WC1E 7HT UK; 2 Guttmacher Institute, 125 Maiden Lane 7th Floor, New York, NY 10038 USA; 3 Population Council, 4 Mwaleshi Road, Lusaka, Zambia; 4 Department of Global Health and Social Medicine, Harvard Medical School, 25 Shattuck St, Boston, MA 02115 USA; 5 Geisel School of Medicine, Dartmouth College, 1 Rope Ferry Rd, Hanover, NH 03755 USA; 6 University of Global Health Equity, Kigali Heights, Plot, 772 Kigali, Rwanda; 7 Makerere University School of Public Health, New Mulago Hill Road, Kampala, Uganda

**Keywords:** Adolescents, Contraception, Family planning, Kenya, Rwanda, Tanzania, Uganda, Private sector

## Abstract

**Background:**

Despite efforts to make contraceptive services more “youth friendly,” unmet need for contraception among young women in sub-Saharan Africa remains high. For health systems to effectively respond to the reproductive health needs of a growing youth population, it is imperative to understand their contraceptive needs and service seeking practices. This paper describes changes over time in contraceptive need, use, and sources of care among young women in four East African countries.

**Methods:**

We used three rounds of DHS data from Kenya, Rwanda, Tanzania, and Uganda to examine time trends from 1999 to 2015 in met need for modern contraception, method mix, and source of care by sector (public or private) and type of provider among young women aged 15–24 years. We assessed disparities in contraceptive coverage improvements over time between younger (15–24 years) and older women (25–49 years) using a difference-in-differences approach.

**Results:**

Met need for contraception among women aged 15–24 years increased over time, ranging from a 20% increase in Tanzania to more than a 5-fold increase in Rwanda. Improvements in met need were greater among older women compared to younger women in Rwanda and Uganda, and higher among younger women in Kenya. Injectables have become the most popular contraceptive choice among young women, with more than 50% of modern contraceptive users aged 15–24 years currently using the method in all countries except for Tanzania, where condoms and injectables are used by 38% and 35% of young users, respectively. More than half of young women in Tanzania and Uganda receive contraceptives from the private sector; however, while the private sector played an important role in meeting the growing contraceptive needs among young women in Tanzania, increased use of public sector services drove expanded access in Kenya, Rwanda, and Uganda.

**Conclusions:**

Our study shows that contraceptive use increased among young East African women, yet, unmet need remains high. As youth populations continue to grow, governments must develop more targeted strategies for expanding access to reproductive health services for young women. Engaging the private sector and task-shifting to lower-level providers offer promising approaches; however, additional research is needed to identify the key facilitators and barriers to the success of these strategies in different contexts.

**Electronic supplementary material:**

The online version of this article (10.1186/s12978-017-0393-3) contains supplementary material, which is available to authorized users.

## Plain English summary

Many young women in sub-Saharan Africa are sexually active and would like to avoid pregnancy, but are not using contraception. The youth population in this region is growing, and it is important for governments to ensure that young people are able to access the reproductive health services that they need. This study used survey data from three time points from 1999 to 2015 in Kenya, Rwanda, Tanzania, and Uganda to describe changes over time in young women’s use of contraceptive services and the types of providers who supplied those services.

We found that the number of young women using contraception is increasing, and the greatest improvements in contraceptive use occurred in Rwanda. Injectable contraceptives have become very popular among young women in all four countries and methods such as condoms and pills are generally less popular. Most of the increases in contraceptive use among young women in Tanzania were due to increased provision of contraceptive services by private for-profit and non-profit health providers. In Kenya, Rwanda, and Uganda, on the other hand, greater use of contraceptives was driven by increased provision of contraceptive services by government health providers.

Although we found that contraceptive use is increasing in East Africa, many young women still do not access the contraceptive services that they need. For governments to keep up with the reproductive health needs of a growing youth population, they must consider where young people are currently seeking contraceptives and develop strategies to further increase access to care.

## Background

Today’s youth population is the largest it has ever been, particularly in sub-Saharan Africa, where nearly 1 in every 3 individuals is aged 10 to 24 years [1]. Despite the size of this population, the health of adolescents and young adults has long been neglected in the global health agenda, in favor of focusing on the health needs of groups such as young children and women of reproductive age more generally [2]. Adolescence is a pivotal period in life, during which many young people begin sexual activity and develop behaviors that could have lasting impact on their health and wellbeing [2]. It is therefore imperative for health systems to respond to these needs and foster positive health-seeking behaviors.

Sub-Saharan Africa has the highest rates of adolescent pregnancy in the world paired with lowest rates of contraceptive use [3–5]. An estimated 1 in 3 adolescent pregnancies in the region are unintended, with over 35% of these unintended pregnancies ending in abortion [5]. Early adolescent pregnancy and childbirth, in particular, has been linked to increased risk of poor health, social, and economic outcomes for young mothers and their children such as anemia during pregnancy, preterm birth, low birthweight, stunting, limited educational and employment opportunities, and poverty [2, 5–10]. Further, complications from childbirth and pregnancy are a leading cause of death among adolescent girls and young women in low- and middle-income countries [11].

For young women in sub-Saharan Africa, access to contraceptive services is critical, yet often unattainable. In many countries, governments and their implementing partners have begun the process of trying to make sexual and reproductive health services more “youth-friendly” [5, 12–16]. However, even where the legal and policy environments are favorable to the reproductive health needs of young people, cultural beliefs, discriminatory practices, and stigma often impede implementation, resulting in persistent barriers to contraceptive access [2, 16–20].

In East and Southern Africa specifically, the rate of teenage pregnancy is particularly high, with an estimated 25% of young women giving birth before the age of 18, compared to an average of 19% in developing countries [4]. Kenya and Tanzania are among the top ten countries in the world with the greatest numbers of young women giving birth by the age of 18 [4]. Further, an estimated 46% and 41% of adolescent births are unplanned in Kenya and Tanzania, respectively [5]. Much of the previous research on youth sexual and reproductive health in this sub-region focuses on knowledge of and attitudes towards contraception, as well as barriers to access. This evidence suggests that although young people are often familiar with contraception, concerns about side effects, fear of stigma, poor provider attitudes towards youth sexual and reproductive health service-seeking, lack of privacy, stock outs, and cost of care prevent them from accessing high quality services even when a need exists [18, 20–35]. Though barriers to care are well documented, less is known about which types of providers are serving the needs of adolescents and young women in East Africa in light of these challenges.

For health systems to effectively respond to the growing population of young people, it is imperative to improve our understanding of their changing reproductive health needs and service-seeking practices, as well as the contexts within which these changes have occurred. The aim of this paper is therefore to describe patterns of contraceptive use, method mix, and sources of contraception among adolescents and young women in four East African countries and discuss the national policies and programs that may have contributed to these patterns. Additionally, in order to assess if population-level improvements in contraceptive access are equitably distributed among younger and older women, we examine if there are differences in the change over time in met need for modern contraception by age group.

## Methods

### Data

This study used cross-sectional, nationally representative Demographic and Health Survey (DHS) data on women aged 15 to 49 years from four countries in the East African Community. Kenya, Rwanda, Tanzania, and Uganda were chosen as case studies to enable comparisons over time, as these were the only countries in the sub-region with at least one DHS survey conducted in each of the following time periods: 1998–2003 (T1), 2004–2009 (T2), and 2010–2015 (T3). In cases where more than one survey was conducted during a time period, the most recent survey was included in the analysis. The surveys included in the analysis are (a) Kenya: 2003, 2008, 2014; (b) Rwanda: 2000, 2005, 2015; (c) Tanzania: 1999, 2005, 2010; and (d) Uganda: 2001, 2006, 2011. We also used demographic data from the United Nations Population Division to estimate absolute numbers of modern contraceptive users over time [36].

### Study population

Our analysis focused on four populations of women aged 15–24 years: (a) all women surveyed, (b) women in need of contraception and (c) women currently using modern contraception (d) women currently using modern contraception from a source whose sector could be classified as public or private. Contraceptive use was also examined among women aged 25–49 years in need of contraception. We refer to women aged 15 to 24 years as youth or younger women and 25 to 49 as older women [37].

### Indicators and definitions

#### Contraceptive need, coverage (met need), and unmet need

In line with the recently revised definition of contraceptive need, women who were not using modern contraception and were either (a) not sexually active (never had sex or not married and have not had sex in the past 30 days), (b) desired to have a child in the next 2 years, or (c) infecund, were considered to not have need for contraception; all others were considered to be in need of contraception for either spacing or limiting [3, 38]. Less than 1% of women in all countries and time periods were missing information on need for contraception; these women were excluded from analyses.

Women were asked whether they were using contraception at the time of survey and where they obtained it the most recent time. We define contraceptive coverage (met need) as the proportion of women in need of contraception who are currently using a modern method. Male and female condoms, pills, injectable contraceptives, implants, the intrauterine device (IUD), male and female sterilization, and other methods such as the diaphragm and foam/jelly were categorized as modern methods. All other methods, including periodic abstinence, withdrawal, and the Lactational Amenorrhea Method were categorized as traditional. Women in need of contraception who were using a traditional method or not using any method at all were considered to have an unmet need for contraception.

#### Method mix

When examining changes in method mix over time, we focused specifically on the four most common currently used methods in the study populations: condoms (male or female), oral contraceptive pills, injectables, and implants. IUDs and sterilization were omitted from analyses as use of either method remained below 2% in all countries and time periods.

#### Source of care by sector

We define source of care as where women received their current method the most recent time. We classified all government providers as public sector and all non-government providers (including for-profit, non-profit, and faith-based providers) as private sector. In cases where it was difficult to determine the sector of the provider, such as contraceptives obtained from husbands, relatives, or friends, or where this information was missing, the sector of care was classified as unknown. Less than 1% of contraceptive users were missing information on source of care, and these are reflected in the “unknown sector” category on figures illustrating contraceptive coverage.

#### Market share by type of provider

We define market share to be the proportion of modern contraceptive users who most recently received their method from a particular source. For this analysis, we excluded women who reported a contraceptive source that could not be classified as either public or private sector. The proportion of modern contraceptive users omitted from market share estimates varied by country and time period (Additional file 1).

Within each sector, there is substantial variation in providers’ skill level, ranging from retailers who can only provide condoms to doctors and nurses who can provide a broad mix of methods, including those requiring more specialized skills such as IUD insertion. Providers were therefore classified by their theoretical capacity to provide a comprehensive mix of both short-term and long-term methods (such as IUD and implants) of modern contraception. Women who received contraception from a source that should typically have the skill level and resources to provide a full method mix (e.g. hospital, clinic, health center) were considered to have received care from a higher capacity, comprehensive provider. In contrast, women who received contraceptive services from a source unlikely to have the skill or resources to offer a full method mix (e.g. community health/outreach worker, pharmacy) were considered to have received care from a limited capacity provider.

### Data analysis

We examined selected socio-demographic characteristics among all women surveyed and women currently using modern contraception disaggregated by age group, country, and survey year (Additional file 2). We calculated the proportion of women aged 15 to 24 years with need, met need, and unmet need for contraception in all four countries over time. Met need was estimated in total and also disaggregated by sector of provision. We estimated unmet need in total and disaggregated by use of traditional methods and use of no method. In order to examine changes in the size of the population in need of contraception, we calculated the absolute number of women aged 15 to 24 years with met and unmet need for contraception by country and period using the mid-year population estimates corresponding to the year that each survey was completed [39]. Among users of public and private sector services, we estimated the proportion of young women most recently receiving care from comprehensive and limited capacity sources. Additionally, we used a Poisson regression model to estimate the change in met need over time by age group and test for the difference-in-differences in time trends in met need between younger and older women [40, 41].

Our analyses were conducted using Stata/SE 14.2. All estimates were appropriately adjusted to take into account survey cluster weights and stratification.

## Results

### Need among all young women

**Fig. 1:**
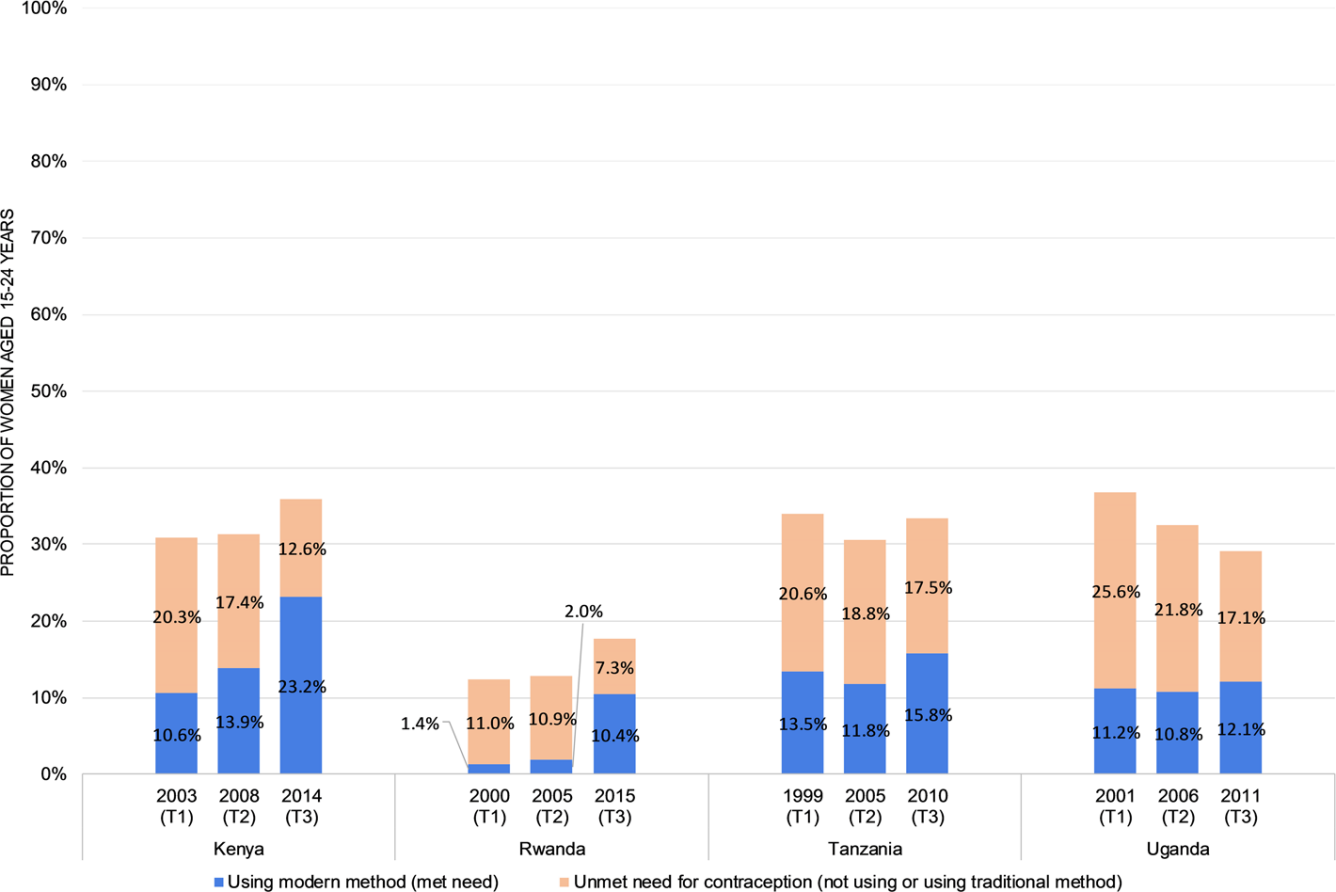
Need for modern contraception among women aged 15–24 years

Figure 1 illustrates the change over time in need for contraception and use of modern methods among women aged 15–24 years in each country. The proportion of young women in need of contraception appears to have increased over time in Kenya and Rwanda; remained fairly constant in Tanzania, and decreased in Uganda. Contraceptive need was consistently lowest in Rwanda, remaining below 18% of young women in all three periods, compared to 30% and above in all other countries and periods.

### Met for modern contraception

**Fig. 2:**
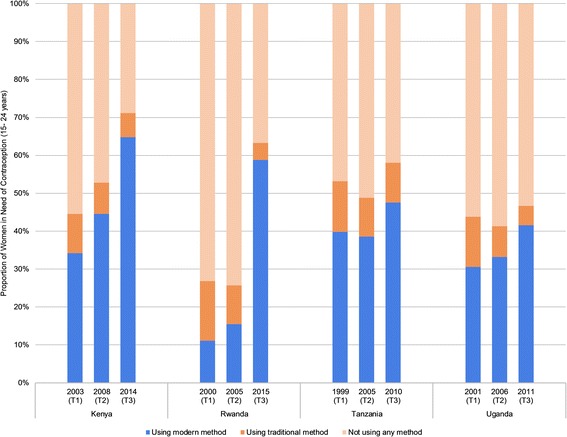
Met need for contraception among women in need aged 15–24 years

Met need among women aged 15–24 years appears to be increasing from T1 to T3 in all four countries (Fig. 2, Additional file 3). With more than a five-fold increase from 11% in 2000 (T1) to 59% in 2015 (T3), Rwanda experienced the most dramatic improvement in met need for contraception among young women (Table 1). Kenya and Uganda experienced smaller but substantial improvements in met need among young women from T1 to T3, increasing from 34% to 65% and 31% to 42%, respectively. Met need appears to have increased over time in Tanzania; however, because the samples of young women in need are quite small, particularly in 1999 (T1), we are unable to determine if this improvement is unique to the women sampled or reflective of population-level trends. Additional file 4 contains a table listing sample sizes for each survey included in the analyses.

**Table 1:** Difference in change in met need over time in younger vs. older women

		Change in met need for family planning from T1 to T3				Difference-in-differences	
Country	Age Group	T1	T3	Relative risk^a^	*p*-value	Relative risk ratio^b^	p-value
Kenya	15–24 years	34.2%	64.8%	1.89	< 0.001	1.38	< 0.001
	25–49 years	52.7%	72.6%	1.38	< 0.001		
Rwanda	15–24 years	11.1%	58.8%	5.30	< 0.001	0.81	0.124
	25–49 years	9.7%	63.4%	6.53	< 0.001		
Tanzania	15–24 years	39.8%	47.5%	1.19	0.141	0.91	0.352
	25–49 years	37.4%	49.2%	1.32	0.001		
Uganda	15–24 years	30.6%	41.5%	1.36	< 0.001	0.82	0.017
	25–49 years	27.7%	45.8%	1.65	< 0.001		

^a^The relative risk compares the met need in T3 to that in T1 for the specified age group

^b^The relative risk ratio compares the relative risk of met need over time for women aged 15–24 years to that of women aged 25–49 years for the specified country

In order to assess whether the improvements in contraceptive coverage differ between younger and older women, we calculated relative risk ratios (RRRs) comparing the change in met need among women aged 15–24 years to the change in met need among women aged 25–49 years for each country. In Kenya, the gap in met need between younger and older women has decreased over time, with younger women experiencing a nearly 40% greater (RRR = 1.38) relative increase in met need compared to older women between 2003 (T1) and 2014 (T3) (Table 1). The observed increases in met need among younger women in Uganda were approximately 18% smaller (RRR = 0.82) than the increases observed among older women. We did not find any evidence of a difference in the relative change in met need over time between younger and older women in Rwanda or Tanzania.

### Method mix

**Fig. 3:**
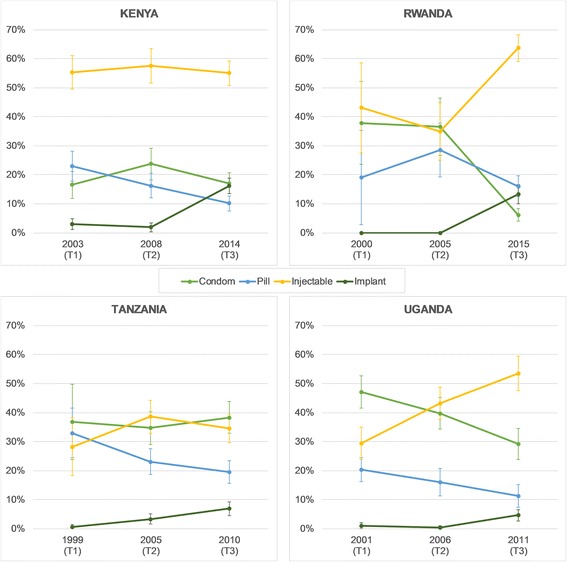
Method mix among current users of modern contraception aged 15–24 years

Figure 3 shows the change in method mix over time among young users of modern contraception by country with 95% confidence intervals displayed for each estimate. Given the small samples of modern contraceptive users aged 15–24 years, the confidence intervals are quite wide, particularly in Rwanda in 2000 (T1).

In Kenya, injectable contraceptives have consistently been the predominant method among women aged 15–24 years, with approximately 55% of current users of modern contraception reporting using injectables in all three study periods. While use of implants increased substantially from less than 5% of users in 2003 (T1) to 16% of users in 2014 (T3), use of pills declined from 23% to 10% of users over the same period. In contrast, condom use remained constant over time, at approximately 17% of modern method users.

Similar to Kenya, injectables have remained popular among young women in Rwanda, with 64% of modern method users aged 15–24 years reporting using the method in 2015 (T3). Use of implants notably increased from no reported users in 2000 (T1) and 2005 (T2) to 13% of modern method users in 2015. In contrast to patterns observed in Kenya, however, condoms as the primary method of contraception declined sharply in Rwanda to below 10% in 2015, while use of pills remained fairly constant over time.

In Tanzania, modern contraceptive users aged 15–24 years were using condoms, injectables, and pills and at similar rates in 1999 (T1), ranging from 28% using injectables to 37% using condoms. Tanzania is the only country where injectables have not penetrated the contraceptive market above 50% of current modern method users in any time period, and where condoms have remained a top method choice, on par with injectables. The pill declined in popularity over time, with less than 20% of young modern method users reporting using the pill in 2010 (T3). Use of the implant increased over time, but remained below 10% of modern method users in all periods.

From 2001 (T1) to 2011 (T3), Uganda experienced major changes in contraceptive method preferences among women aged 15–24 years, with use of condoms as a primary method of contraception declining and reported use of injectables increasing to more than half of modern method users. While pills appear to have lost popularity, implant use increased over time, but remained below 5% of modern method users.

### Coverage and source of care by sector

**Fig. 4:**
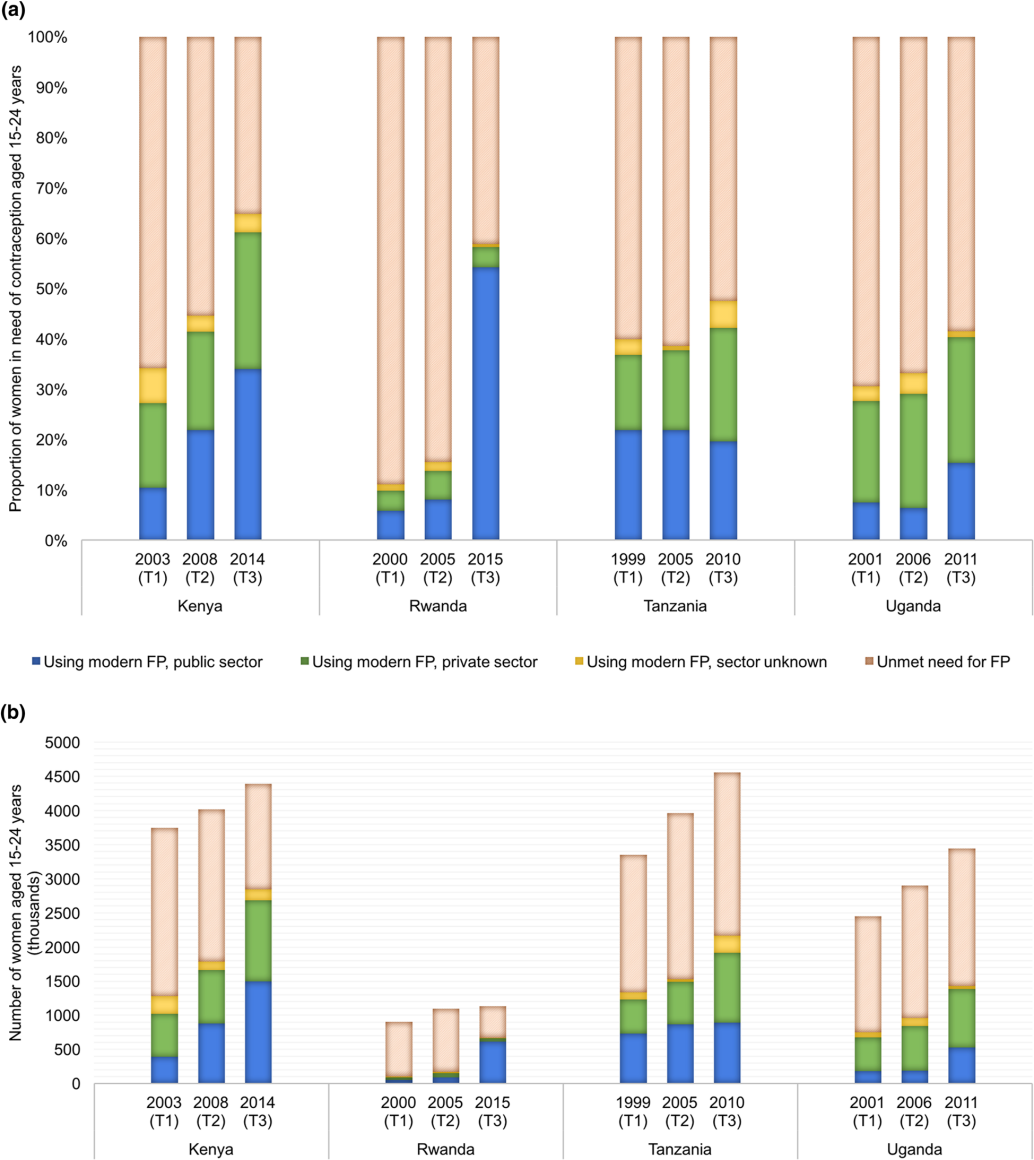
Contraceptive use and source of care among women aged 15–24 years. **a** Source of care among women in need in contraception aged 15-24 years. **b** Absolute need and source of care aged 15-24 years

We found substantial variation between countries in levels and patterns of use of public sector contraceptive services. The proportion of contraceptive need met by the public sector in Kenya grew steadily from 10% in 2003 (T1) to 34% in 2014 (T3) (Fig. 4a, Additional file 3). Gains in public sector coverage occurred primarily between T2 and T3 in Rwanda and Uganda, increasing from 8% to 54% in Rwanda and 6% to 15% in Uganda. Public sector contraceptive coverage in Tanzania, on the other hand, remained relatively constant over time, between 20 and 22% in all three study periods.

At T1, the private sector in Kenya, Tanzania, and Uganda covered a sizable portion of contraceptive need among women aged 15–24 years, ranging from 15% in Tanzania to 20% in Uganda. By T3, the private sector had grown in all three countries, providing contraceptive services to approximately 1 in 4 young women in need. Private sector coverage of contraceptive need in Rwanda, in contrast, has remained below 6% in all three periods.

While Fig. 4a illustrates the *proportion* of young women with a met need for contraception by sector, Fig. 4b depicts the estimated *number* of women served, taking into account increases in absolute population size over time. We found that despite Rwanda’s tremendous improvements in the proportion of women aged 15–24 years with a met need for contraception, the absolute increase in the number of modern contraceptive users in Rwanda was very small in comparison to the increases in population coverage observed in the other three countries (Fig. 4b). For instance, though in relative terms the growth of public sector was much larger in Rwanda than in Kenya, on an absolute scale, the public sector in Kenya provided contraception to approximately 1.1 million additional users between 2003 (T1) and 2014 (T3), while the public sector in Rwanda served half as many additional users from 2000 (T1) to 2015 (T3) (Fig. 4b).

### Market share by provider type

**Fig. 5:**
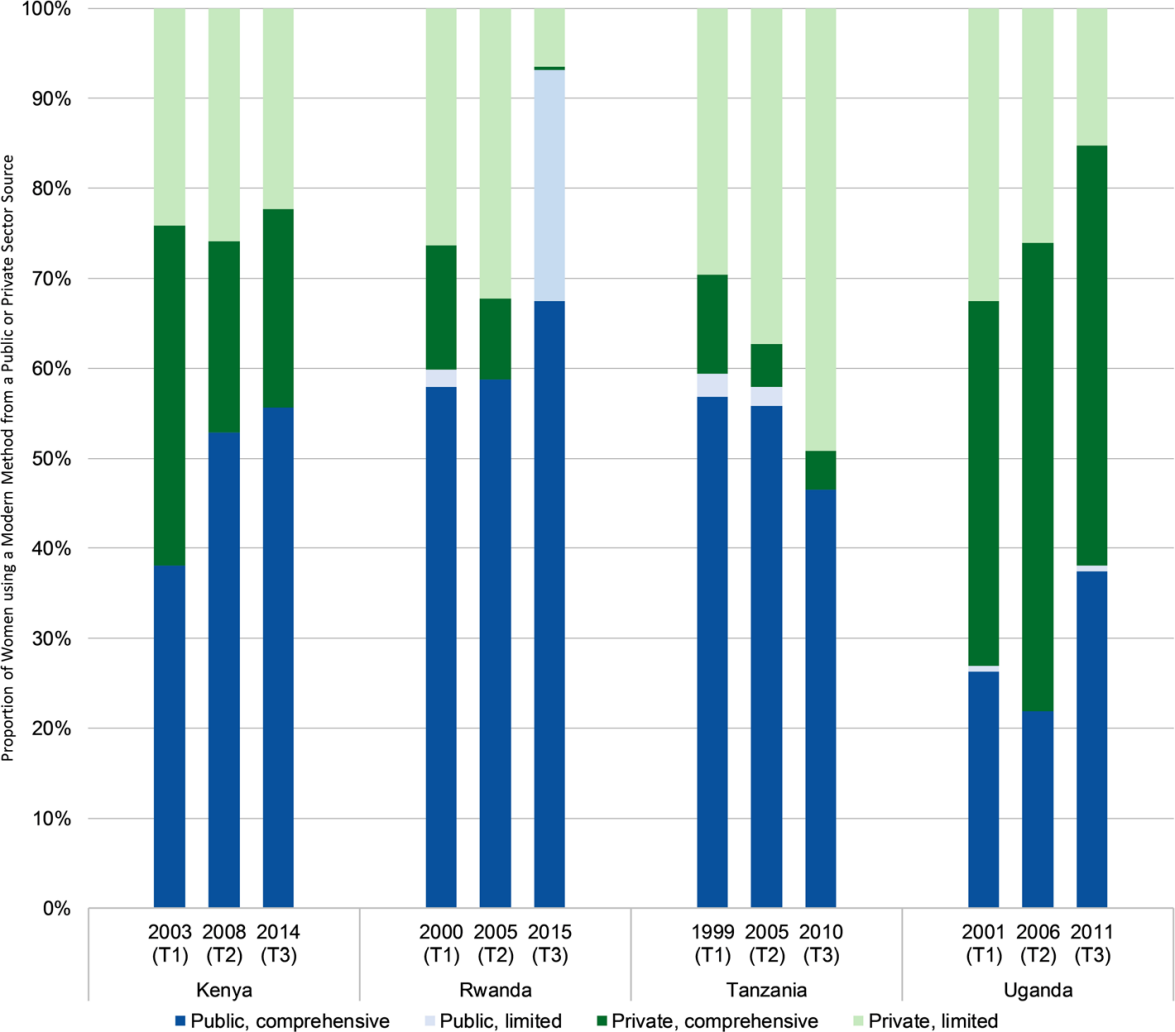
Provider type among young women using modern contraception from public or private sector source

Notable changes occurred over time in the types of providers visited by young users of public and private sector contraceptive services (Fig. 5). In Kenya, Tanzania, and Uganda, public sector services were provided nearly exclusively by higher capacity comprehensive providers during all three study periods. While limited capacity providers played a similarly small role in public sector contraceptive provision in Rwanda in 2000 (T1) and 2005 (T2), the growth of the public sector between 2005 and 2015 (T3) was largely driven by increased use of limited capacity sources, specifically, government community health workers.

The market share for comprehensive private sector providers such as private hospitals and non-governmental clinics has decreased over time in Kenya; however, they remain an important source of contraception for young Kenyan women. In contrast, use of comprehensive private providers has diminished in Rwanda and Tanzania, decreasing to below 5% in both countries. In Uganda, comprehensive private facilities have remained the most popular contraceptive provider, with more than 40% of young women receiving care from these providers in all three periods.

In all four countries at T1, between 25% and 33% of modern contraceptive users aged 15–24 years reported most recently receiving their method from a limited capacity private sector provider such as a drug seller or commercial shop. Market share for limited capacity private sector providers remained relatively constant in Kenya and decreased in Rwanda and Uganda to 7% and 15%, respectively (Fig. 5). In contrast, market share for limited capacity private sector providers increased over time in Tanzania to nearly half of modern contraceptive users aged 15–24 years.

## Discussion

We used data from three rounds of DHS surveys in Kenya, Rwanda, Tanzania, and Uganda to explore level of need, use of contraception, method mix, and sources of care among women aged 15–24 years. This is the first study to comprehensively examine how the source of contraceptive services for young women in Kenya, Rwanda, Tanzania, and Uganda has changed over time given the growth in the youth population and the aforementioned constraints to contraceptive service-seeking. Our findings show that the proportion of youth with met need has increased in the sub-region, and this is a particularly notable accomplishment given the substantial population growth over time. Overall, young women appear to be shifting away from condoms and pills and instead opting for injectable contraceptives. Use of implants remains low but increasing in all four countries. In Kenya, Uganda and Tanzania, the private sector has remained an important provider of  contraceptive services among youth. In contrast, the contribution of the private sector has declined and most of the increase in met need in Rwanda appears to have been achieved via the expansion of government community health workers. These findings are promising; however, despite the progress that has been achieved, a substantial unmet need for contraception among young women East Africa still remains, ranging from 35% in Kenya to 59% in Uganda. Governments in these countries will need to develop more effective and targeted strategies to sufficiently increase access to high-quality contraceptive services to meet both the rising youth population and need for sexual and reproductive health services.

### Pathways to increased contraceptive coverage

Our cross-country comparative study enabled us to uncover the different paths countries took towards increased contraceptive coverage among youth, and consider their accomplishments within the contexts in which they occurred.

Kenya was the only country where the increase in access to contraception among women aged 15–24 years outpaced changes in contraceptive coverage over time among women aged 25–49 years. Kenya’s progress in closing the gap in met need between younger and older women occurred in a supportive policy environment. For instance, the Government of Kenya has established multiple guidelines and policies across sectors that support the sexual and reproductive health rights for young people, including, but not limited to, the National Adolescent Reproductive Health Development Policy (2003); Guidelines for the Provision of Youth Friendly Services (2005); Gender Policy in Education (2007); National Youth Policy (2007); Ministry of Youth Affairs Strategic Plan (2007), National Reproductive Health Strategy (2009); and the National Adolescent Sexual and Reproductive Health Policy (2015) [42–46]. Additionally, several interventions aimed at increasing demand for and access to sexual and reproductive health services among youths have been implemented in Kenya, including youth-friendly health services, safe spaces, mass media campaigns, and entertainment and sports-centered activities [31, 47–49]. While there is ample evidence of these approaches being tested in Kenya, it is unclear the extent to which these efforts represent a scalable, coordinated policy-driven response that could be adapted to other settings [43, 47].

The increase in met need over time among young women was exceptionally large in Rwanda compared to Kenya, Tanzania, and Uganda. Rwanda’s small population size, high population density, and low levels of need compared to these countries may have contributed to the government’s success in greatly expanding use of contraception in a short period of time [36]. The bulk of the improvements in coverage occurred between 2005 and 2015, and this coincides with implementation of the Government of Rwanda’s 2008–2012 poverty reduction strategy, which prioritized limiting population growth not only as a health or human rights issue, but also as a critical component for increased economic development [50, 51]. One strategy that has contributed tremendously to expanding access to contraceptives in Rwanda is the training of government-supported community health workers to offer comprehensive contraceptive counseling and provide short-term methods at the community level, including condoms, pills, and injectables [52, 53]. Additionally, high coverage of Rwanda’s community-based health insurance program, Mutuelles de Santé, likely helped to reduce financial barriers associated with contraceptive service seeking following its scale-up in 2006 [54, 55]. Though these strategies have been effective at increasing access to contraception overall, unmet need remains greater among younger women compared to older women. Rwanda’s first Adolescent and Reproductive Health and Rights Policy, enacted in 2012, outlines plans to decentralize sexual and reproductive health services, make them more youth-friendly, and strengthen the role of the private sector in service provision [56]. The observed disparities between younger and older women suggest that broader social and cultural barriers may be inhibiting the implementation and scale-up of this policy in practice [57]. It is important to better understand these barriers to contraceptive access for young women in Rwanda and consider whether engaging the private sector, which currently only covers 5% of contraceptive need among women aged 15 to 24 years, might offer an appropriate approach for expanding access.

The public sector played an important role in increasing young women’s access to contraceptive services in Kenya, Rwanda, and Uganda, as growth in public sector coverage outpaced that in the private sector. In contrast, the increase in met need in Tanzania was driven by expanded access in the private sector. Most young women in Kenya and Rwanda now receive their contraception from a public sector source, while more than half of women aged 15–24 years in Tanzania and Uganda receive their care from the private sector. Given the challenges that many governments may face in trying to regulate their expansive and diverse private sectors, this finding raises questions about the quality of care and out-of-pocket expenditures young women experience when seeking contraceptive services [3, 58]. Nearly half of all young contraceptive users in Tanzania received their method from a limited capacity provider such as a drug seller or retail shop in 2010, and this may be related to the high use of condoms. Another contributing factor might be the Tanzanian government’s collaboration with private sector vendors to increase access to high quality medicines, including reproductive health commodities, under the accrediting drug dispensing outlet (ADDO) program [59]. While this type of initiative may help to increase access, particularly where access to health facilities is limited, evaluations of other programs aimed at improving the quality of care received at drug sellers in Ghana, Nigeria, Tanzania, and Uganda have shown improvements in drug seller knowledge, but inconsistent evidence of improved practices such as counseling and provision of appropriate drugs [60].

Previous studies conducted in Tanzania and Uganda have found that private sector facilities are more likely to have stock outs of contraceptive methods and less likely to provide a comprehensive mix of short- and long-term methods compared to public sector facilities [35, 61–66]. Further, two studies on young people’s perceptions of reproductive health services in Uganda identified stock outs and inaccessibility of contraceptive commodities as key barriers to contraceptive use [18, 26]. The fact that unmet need among young women is highest in Tanzania and Uganda (53% and 59%, respectively, on most recent surveys), where private sector market share is also highest raises questions as to whether the private sector is complementing governments’ efforts by reaching young women who otherwise may not have access to contraceptive services, or perhaps whether the private sector is serving as a replacement for government services, which are in some way less accessible to young people.

In terms of method mix, injectables are a popular choice among women aged 15–24 years in Kenya, Rwanda, Tanzania, and Uganda; however, longer-term methods such as the implant and IUD have not gained much traction with young women over the years. Due to the young age of these women, it is likely that a substantial proportion wish to delay or space their births for two or more years, but perhaps have infrequent sex, which might help explain their preference for shorter-term methods that are easier to start and stop as needed [67, 68]. Injectables offer an appealing option for young people, as they provide shorter-term protection compared to long-acting methods, but require less frequent use compared to condoms and pills. Low uptake of implants and IUDs may also relate to other barriers such as lack of availability, costs, and fears, misconceptions, and provider biases.

Tanzania is the only one of the study countries where condoms have remained the most popular method of contraception among young women. Use of condoms as a primary form of contraception has decreased Rwanda and Uganda, reaching as low as 6% of modern contraceptive users aged 15–24 years in Rwanda. While it is favorable for young people to adopt more effective contraceptive methods, this shift away from condoms may also pose serious risks with regard to the prevention of sexually-transmitted infections (STIs). For instance, more than half of never-married sexually active adolescent women in Rwanda report not using a condom during their last sexual encounter [5]. Given that all four countries are affected by the HIV epidemic, it is critical to ensure that as young people shift towards more effective contraceptives, they are regularly counseled about the importance of dual protection with a condom and a non-barrier method of contraception to prevent both unintended pregnancy and STIs, including HIV [69, 70].

### Limitations

Our study has some limitations resulting from the use of DHS data. First, the analysis relies on women’s self-reports on their sexual and reproductive health needs and practices. Unmet need is estimated based on self-reported sexual activity in the last 30 days, which is likely to be underreported, particularly among young, unmarried women [71]. It is therefore likely that we underestimate unmet need for contraception and overestimate met need. Additionally, although we compare time trends in four countries across three periods, the surveys were not conducted during the same years in all countries. For instance, the T3 surveys in Tanzania and Uganda were conducted in 2010 and 2011, while the surveys in Kenya and Rwanda were conducted in 2014 and 2015, respectively. What appears to be slower progress in increasing young women’s access to contraception in Tanzania and Uganda, therefore, may be due to their earlier survey dates.

Determining source of care in terms of sector and capacity of provider is also challenging due to both self-reporting and survey response options. Faith-based providers have an undeniable presence in sub-Saharan Africa; however, the extent of their contribution to contraceptive service provision is less certain [72–74]. Accurately reporting sector of care can be challenging for women, particularly in cases where faith-based and other non-governmental organizations are closely aligned with public sector service provision. We were therefore limited in our ability to accurately disaggregate the contributions of different types of private sector providers. Distinguishing provider type is also difficult due to conflation of response options on the survey [75]. For instance, all three surveys from Tanzania had the response option “public government dispensary/pharmacy.” Dispensaries in Tanzania are equivalent to small clinics or health posts in other countries and would therefore be classified as a comprehensive contraceptive provider. Pharmacies, on the other hand, would be considered a limited capacity provider. Further, it is important to note that the theoretical capacity of a provider to offer both short- and long-acting methods does not always reflect practice.

This analysis of young women’s contraceptive need and use was also limited by data availability for younger adolescents, as the DHS only interviews women aged 15 years and above. Although a number of young women in our study countries report beginning sexual activity before the age of 15 years, very little evidence exists on contraceptive needs and use among younger adolescents aged 10–14 years [2, 76].

## Conclusions

Our findings show an increasing number and proportion of young women are using contraceptive services in East Africa. Despite these improvements in contraceptive access, a substantial proportion of the population is still not accessing these services. As the adolescent and young adult populations in these countries are projected to continue growing over the next several decades, it is critical for governments to develop more effective strategies for rapidly expanding access to high quality contraceptive services for youth and eliminating any existing disparities in met need between younger and older women [1, 2]. Based on the experiences of these four countries, engaging with the private sector and task-shifting to lower-level government providers such as community health workers may offer two promising approaches to increasing access to contraceptive services for youth, provided services are appropriately regulated and minimum quality standards are maintained. However, each country is unique and will need to adapt these strategies to their particular contexts. Further in-depth research into the packages of interventions and contextual factors that contributed to the observed trends would help countries to identify key facilitators and barriers to achieving universal access to reproductive health services for young people.

## Additional files

Additional file 1: Missing information on sector of care among current users of modern contraception aged 15–24 years. (DOCX 14 kb)

Additional file 2: Socio-demographic characteristics of all women surveyed and current users of modern contraception. (XLSX 22 kb)

Additional file 3: Contraceptive unmet need, use, sector of care, and provider type by country and period. (DOCX 15 kb)

Additional file 4: DHS datasets & weighted sample sizes for populations included in analysis. (DOCX 16 kb)

## Abbreviations

DHS: Demographic and Health Survey
IUD: Intrauterine device
STI: Sexually-transmitted infection
