# Supplementary material for: Pathways to increased coverage: an analysis of time trends in contraceptive need and use among adolescents and young women in Kenya, Rwanda, Tanzania, and Uganda
Source: Reprod Health. 2017 Oct 17;14:130. doi: 10.1186/s12978-017-0393-3 (PMC5645984; doi:10.1186/s12978-017-0393-3)
Supplement: Supplementary file 1 — Missing information on sector of care among current users of modern contraception aged 15–24 years. (DOCX 14 kb) [file 12978_2017_393_MOESM1_ESM.docx]

**Table S1:** Missing information on sector of care among current users of modern contraception aged 15-24 years

|  |  | **Omitted Observations** | |
| --- | --- | --- | --- |
| **Country/Year** | **Users of modern FP (N)** | **Unidentifiable sector (%)** | **Sector missing**  **(%)** |
| **Kenya** |  |  |  |
| 2003 | 375 | 4.01 % | 16.29 % |
| 2008 | 485 | 5.73 % | 1.40 % |
| 2014 | 1256 | 4.68 % | 0.82 % |
| **Rwanda** |  |  |  |
| 2000 | 61 | 11.27 % | 0.00 % |
| 2005 | 97 | 2.55 % | 8.67 % |
| 2015 | 544 | 0.43 % | 0.56 % |
| **Tanzania** |  |  |  |
| 1999 | 232 | 7.03 % | 0.58 % |
| 2005 | 500 | 2.25 % | 0.00 % |
| 2010 | 646 | 3.56 % | 7.79 % |
| **Uganda** |  |  |  |
| 2001 | 350 | 9.02 % | 0.55 % |
| 2006 | 393 | 11.04 % | 1.35 % |
| 2011 | 444 | 2.88 % | 0.00 % |
