# Supplementary material for: Pathways to increased coverage: an analysis of time trends in contraceptive need and use among adolescents and young women in Kenya, Rwanda, Tanzania, and Uganda
Source: Reprod Health. 2017 Oct 17;14:130. doi: 10.1186/s12978-017-0393-3 (PMC5645984; doi:10.1186/s12978-017-0393-3)
Supplement: Supplementary file 3 — Contraceptive unmet need, use, sector of care, and provider type by country and period. (DOCX 15 kb) [file 12978_2017_393_MOESM3_ESM.docx]

| **Country** | **Year of Survey** | **15-24 years** | | |  | **25-49 years** | | |
| --- | --- | --- | --- | --- | --- | --- | --- | --- |
|  |  | **Total**  **Sample** | **In need of contraception** | **Currently using a modern method** |  | **Total**  **Sample** | **In need of contraception** | **Currently using a modern method** |
| Kenya | 2003 | 3547 | 1096 | 375 |  | 4648 | 2824 | 1487 |
| Kenya | 2008 | 3475 | 1088 | 485 |  | 4969 | 3133 | 1844 |
| Kenya | 2014 | 5403 | 1939 | 1,256 |  | 9212 | 6219 | 4512 |
| Rwanda | 2000 | 4524 | 551 | 61 |  | 5897 | 2229 | 216 |
| Rwanda | 2005 | 4938 | 626 | 97 |  | 6383 | 2822 | 469 |
| Rwanda | 2015 | 5225 | 925 | 544 |  | 8272 | 4938 | 3130 |
| Tanzania | 1999 | 1720 | 581 | 232 |  | 2309 | 1064 | 398 |
| Tanzania | 2005 | 4252 | 1297 | 500 |  | 6077 | 3034 | 1279 |
| Tanzania | 2010 | 4081 | 1359 | 646 |  | 6058 | 3354 | 1650 |
| Uganda | 2001 | 3119 | 1147 | 350 |  | 4127 | 2228 | 618 |
| Uganda | 2006 | 3646 | 1186 | 393 |  | 4885 | 2780 | 922 |
| Uganda | 2011 | 3677 | 1071 | 444 |  | 4997 | 2922 | 1339 |

**Table S3: DHS datasets & weighted sample sizes for populations included in analysis**
