# Supplementary material for: Pathways to increased coverage: an analysis of time trends in contraceptive need and use among adolescents and young women in Kenya, Rwanda, Tanzania, and Uganda
Source: Reprod Health. 2017 Oct 17;14:130. doi: 10.1186/s12978-017-0393-3 (PMC5645984; doi:10.1186/s12978-017-0393-3)
Supplement: Supplementary file 4 — DHS datasets & weighted sample sizes for populations included in analysis. (DOCX 16 kb) [file 12978_2017_393_MOESM4_ESM.docx]

| **Country** | **Year of Survey** | **Women aged 15-24 years in need of contraception** | | | | |  | **Women aged 15-24 years using public or private sector services by provider type** | | | |
| --- | --- | --- | --- | --- | --- | --- | --- | --- | --- | --- | --- |
|  |  | **Unmet need** | **Met need** | **Using modern method, public sector** | **Using modern method, private sector** | **Using modern method, sector unknown** |  | **Public comprehensive** | **Public limited** | **Private comprehensive** | **Private limited** |
| Kenya | 2003 | 65.8% | 34.2% | 10.4% | 16.9% | 6.9% |  | 38.1% | 0.0% | 37.7% | 24.1% |
|  | 2008 | 55.4% | 44.6% | 21.9% | 19.5% | 3.2% |  | 52.8% | 0.0% | 21.3% | 25.9% |
|  | 2014 | 35.2% | 64.8% | 34.1% | 27.1% | 3.6% |  | 55.6% | 0.0% | 22.0% | 22.3% |
| Rwanda | 2000 | 88.9% | 11.1% | 5.9% | 4.0% | 1.2% |  | 57.9% | 1.9% | 13.8% | 26.4% |
|  | 2005 | 84.5% | 15.5% | 8.1% | 5.7% | 1.7% |  | 58.8% | 0.0% | 9.0% | 32.3% |
|  | 2015 | 41.2% | 58.8% | 54.2% | 4.0% | 0.6% |  | 67.4% | 25.6% | 0.4% | 6.5% |
| Tanzania | 1999 | 60.2% | 39.8% | 21.9% | 15.0% | 3.0% |  | 56.8% | 2.6% | 11.0% | 29.6% |
|  | 2005 | 61.5% | 38.5% | 21.8% | 15.9% | 0.9% |  | 55.8% | 2.1% | 4.8% | 37.3% |
|  | 2010 | 52.5% | 47.5% | 19.6% | 22.5% | 5.4% |  | 46.5% | 0.0% | 4.3% | 49.2% |
| Uganda | 2001 | 69.4% | 30.6% | 7.4% | 20.2% | 2.9% |  | 26.3% | 0.6% | 40.5% | 32.6% |
|  | 2006 | 66.8% | 33.2% | 6.4% | 22.7% | 4.1% |  | 21.9% | 0.0% | 52.0% | 26.1% |
|  | 2011 | 58.5% | 41.5% | 15.3% | 25.0% | 1.2% |  | 37.4% | 0.7% | 46.7% | 15.2% |

**Table S4: Contraceptive unmet need, use, sector of care, and provider type by country and period**
